# A Survey of Culturable Fungal Endophytes From *Festuca rubra* subsp. *pruinosa*, a Grass From Marine Cliffs, Reveals a Core Microbiome

**DOI:** 10.3389/fmicb.2018.03321

**Published:** 2019-01-16

**Authors:** Eric Pereira, Beatriz R. Vázquez de Aldana, Leticia San Emeterio, Iñigo Zabalgogeazcoa

**Affiliations:** ^1^Institute of Natural Resources and Agrobiology of Salamanca, Consejo Superior de Investigaciones Científicas (IRNASA-CSIC), Salamanca, Spain; ^2^Research Institute on Innovation & Sustainable Development in Food Chain (ISFood), Universidad Pública de Navarra, Pamplona, Spain

**Keywords:** mycobiome, *Diaporthe*, Fusarium oxysporum, *Epichloë*, salinity, halophyte, grass

## Abstract

*Festuca rubra* subsp. *pruinosa* is a perennial grass that inhabits sea cliffs of the Atlantic coasts of Europe. In this unhospitable environment plants grow in rock crevices and are exposed to abiotic stress factors such as low nutrient availability, wind, and salinity. *Festuca rubra* subsp. *pruinosa* is a host of the fungal endophyte *Epichloë festucae*, which colonizes aerial organs, but its root mycobiota is unknown. The culturable endophytic mycobiota of FRP roots was surveyed in a set of 105 plants sampled at five populations in marine cliffs from the northern coast of Spain. In total, 135 different fungal taxa were identified, 17 of them occurred in more than 10% of plants and in two or more populations. Seven taxa belonging to *Fusarium*, *Diaporthe*, Helotiales, *Drechslera*, *Slopeiomyces*, and *Penicillium* appeared to be constituents of the core microbiome of *Festuca rubra* subsp. *pruinosa* roots because they occurred in more than 20% of the plants analyzed, and at three or more populations. Most fungal strains analyzed (71.8%) were halotolerant. The presence of *Epichloë festucae* in aboveground tissue was detected in 65.7% of the plants, but its presence did not seem to significantly affect the structure of the core or other root microbiota, when compared to that of plants free of this endophyte. When plants of the grass *Lolium perenne* were inoculated with fungal strains obtained from *Festuca rubra* subsp. *pruinosa* roots, a *Diaporthe* strain significantly promoted leaf biomass production under normal and saline (200 mM NaCl) watering regimes. These results suggest that the core mycobiome of *Festuca rubra* subsp. *pruinosa* could have a role in host plant adaptation, and might be useful for the improvement of agricultural grasses.

## Introduction

The vegetation that inhabits coastal marine cliffs is adapted to environmental conditions that are far from optimal for plant growth and survival. The rock substrate and vertical cliffs makes soil scarce or non-existent. Sea water spray adds salinity to the scenario, and exposure to sea winds favor plant dehydration. Those conditions of low nutrient availability, salinity, and wind exposure can be persistent in sea cliffs, and as a result, sea cliff vegetation is often endemic, reflecting habitat specialization in order to survive under these unhospitable conditions (Doody, 2001; López-Bedoya and Pérez-Alberti, 2009).

*Festuca rubra* subsp. *pruinosa* (FRP) is a plant species common in cliffs of the Atlantic coasts of Europe (Markgraf-Dannenberg, 1980; López-Bedoya and Pérez-Alberti, 2009). This perennial grass grows as a chasmophyte in rock fissures, or in very shallow soils formed on cliff cavities and slopes. In nature this species rarely occurs away from sea cliffs, where other vegetation predominates, and its salt tolerance is greater than that of other *F. rubra* subspecies adapted to inland habitats (Humpreys, 1982). Some anatomical characteristics might contribute to the adaptation to cliffs of this plant, for instance, the epithet *pruinosa* refers to the apparent epicuticular wax coat that covers its leaves, possibly having a role in preventing water loss (Ortuñez and de la Fuente, 2010; Martínez Sagarra et al., 2017).

In addition to traits inherent to the plant genome, the plant microbiome can also contribute to adaptation. Studies of some plants adapted to high stress habitats revealed that fungal endophytes confer habitat-specific stress tolerance to their hosts, and without these fungal endophytes plant adaptation is reduced in their native habitats (Rodriguez and Redman, 2008). Examples include improved tolerance to biotic and abiotic stress factors such as disease, herbivory, heat, or salinity mediated by endophytic fungi (Clay and Schardl, 2002; Waller et al., 2005; Rodriguez et al., 2008). Some of the endophytes reported in these studies conferred improved stress tolerance to new host species, highlighting the importance that endophytic fungi could have for the improvement of agricultural crops.

Like other subspecies of *Festuca rubra*, FRP plants maintain associations with the fungal endophyte *Epichloë festucae*. This fungus systemically colonizes the stems and leaves of host plants, but not the roots, and it is transmitted vertically to seeds (Leuchtmann et al., 1994; Zabalgogeazcoa et al., 2006). Endophytic *Epichloë* species can have a mutualistic relationship with their hosts, and increased tolerance of symbiotic plants to biotic and abiotic stress factors have been reported to occur in some situations. For example, *Epichloë festucae* can produce several types of alkaloids that might protect host plants against herbivores (Clay and Schardl, 2002).

In marine cliffs the roots of FRP plants grow in rock fissures or minimal soil, forming a compact fibrous system which holds the plant and captures nutrients. The root mycobiota of FRP is unknown, and some of its components could be useful for the improvement of other plant species of agronomic interest, as it has been demonstrated in other plant-endophyte associations (Rodriguez et al., 2008). Thus, the objectives of this work were: (1) to identify the culturable endophytic mycobiota of FRP roots, (2) to determine if the presence of *Epichloë* affects the structure of the root mycobiota, and (3) to test if some FRP root endophytes affect the performance of another grass, *Lolium perenne*, when exposed to salinity.

## Materials and Methods

### Study Sites and Plant Sampling

Plants of *Festuca rubra* subsp. *pruinosa* (FRP) were collected at five locations in sea cliffs in the North Atlantic coast of Spain. Three locations were in Galicia: Torre de Hércules (TDH), 43°23′09^′′^N 8°24′23^′′^W, Cedeira (CED), 43°40′46^′′^N 8°01′15^′′^W, and Estaca de Bares (EDB), 43°47′25^′′^N 7°41′16^′′^W, and two in Asturias: San Pedro de la Rivera (SPR), 43°34′43^′′^N 6°13′17^′′^W, and Cabo de Peñas (CDP), 43°39′02^′′^N 5°51′00^′′^W. The shortest distance in straight line among these locations is 30 km. The predominant flora in the walls of these sea cliffs mainly consisted of *Festuca rubra* subsp. *pruinosa*, *Armeria* spp. and *Crithmum maritimum*. The climate in the coast of Galicia and Asturias is mild with oceanic influence and abundant rainfall spread over the year; during the 1981–2010 period the mean annual precipitation was 1106 and 1062 mm, and the average annual temperature 13.5 and 13.8°C in Galicia and Asturias, respectively (AEMET, 2012). In the spring of 2016, a total of 105 FRP plants, about 20 plants per location, were collected. Most plants grew in fissures in the rock, where soil was very scarce or absent. The plants were transported in a refrigerated cooler to the laboratory in Salamanca, and processed for the isolation of fungi from roots the day after they were sampled. Afterward the plants were transplanted to pots with a 1:1 (v:v) mixture of peat and perlite and maintained in a wirehouse outdoors.

### Isolation of Fungi

To isolate fungi from roots, a sample of about 20 root fragments of 4–5 cm was collected from each plant. Each root sample was surface-disinfected with a solution of 20% commercial bleach (1% active chlorine) containing 0.02% Tween 80 (v:v) for 6 min, followed by treatment with an aqueous solution of 70% ethanol for 30 s. Finally, the roots were rinsed with sterile water and cut into pieces about 5 mm long. Thirty root pieces of each sample were plated in two Petri plates (15 pieces/plate) with potato dextrose agar (PDA) containing 200 mg/L of chloramphenicol. This antibiotic was used to exclude the isolation of endophytic bacteria. A root sample of each of the 105 plants was prepared as outlined above, and kept in the dark at room temperature. As mycelium emerged from a root fragment into the agar, a small piece of the mycelium from the leading edge of the colony was transferred to a new PDA plate and maintained at room temperature. The root fragment and remaining mycelium were taken out of the original plate to avoid overgrowth. The plates with root samples were checked daily for the presence of fungi for about 4 weeks.

The presence of *Epichloë festucae* on each plant was diagnosed by isolation. Several leaf sheaths were collected from each plant, cut into fragments about 5 mm long, and surface disinfected by immersion in a solution of 20% commercial bleach for 10 min. The fragments were then rinsed with sterile water, and about 15 fragments from each plant were placed in a PDA plate containing 200 mg/L of chloramphenicol. The plates were kept at room temperature, and fungi emerging from leaf fragments during the first 2–5 days were discarded together with its leaf sheath fragment. White *Epichloë* mycelium emerging from the extremes of the leaf fragments about 1 week after plating was transferred to new PDA plates for further identification.

### Identification of Fungi

The fungal isolates obtained from roots were first grouped into different morphotypes according to morphological characteristics such as colony color, exudate production, mycelium appearance, and growth rate. One or a few isolates of each morphotype were used for further classification based on rDNA nucleotide sequences. Fungal DNA was extracted from a small amount of mycelium scraped from a PDA culture using the Phire Plant Direct PCR Kit (Thermo Fisher Scientific). A ribosomal DNA region including the internal transcribed spacer 1 (ITS1), 5.8S rDNA, and ITS2 was amplified by PCR using primers ITS1 and ITS4 (White et al., 1990). Amplification conditions were: 98°C for 5 min, followed by 35 cycles of 98°C for 5 s, 54°C for 5 s, and 72°C for 20 s; after that the reaction was kept at 72°C for 1 min. PCR amplicons were cleaned (MSB Spin PCRapace, Stratec biomedical, Germany) and sequenced at the DNA sequencing service of the University of Salamanca (Spain).

All the sequences obtained were grouped into operational taxonomic units (OTU), considering that groups of sequences with a similarity greater than 97% belonged to the same OTU. This clustering operation was done using BlastClust software (NCBI, 2004). Afterward, a sequence representative of each OTU was used to search for similar curated sequences at the UNITE fungal database. A taxonomic identity was assigned to each OTU considering that the species rank of a UNITE database match was accepted when the identity between the OTU and database sequences was greater than 97%, and most UNITE matches corresponded to the same taxon. When the similarity was 97% – 95%, or UNITE matches corresponded to several species of the same genus, only the genus rank was accepted. In other cases the sequences were assigned to orders or families whenever it was reasonable.

### Analysis of Root Fungal Diversity

For each location (referred to as population from here on), species accumulation curves showing the relationship between the number of plants sampled and the number of fungal species obtained, were estimated using the ‘specaccum’ function and the exact method with the Vegan Package in R (Oksanen et al., 2017). Estimations of the maximum number of fungal species at each population were obtained with the Bootstrap and Chao indexes using EstimateS 9.0 software (Colwell, 2005). Shannon’s index of diversity (H’) was estimated from the relative abundance of each taxon identified. The distribution of the relative abundance of the fungal species was observed with a rank-abundance curve. The similarity of fungal communities between each pair of populations was estimated using Jaccard’s index of similarity (J). It is calculated from the equation J = c/(a + b + c), where ‘c’ is the number of fungal taxa shared between two populations, ‘a’ the number of fungal taxa unique to the first population and ‘b’ the number of fungal taxa unique to the second population (Jaccard, 1912).

### Effect of *Epichloë* on Root Mycobiota

Species richness (number of different root endophyte species per plant) was analyzed with a two-way ANOVA with *Epichloë* presence (E+) or absence (E-) and plant population (CED, CDP, EDB, SPR, and TDH) as factors. A type III sum of squares was used because the number of E+ and E- plants was unbalanced.

Species accumulation curves and beta diversity index estimations, plus a Canonical Correspondence Analysis (CCA) were made using the Vegan Package in R (Oksanen et al., 2017). Species accumulation curves for E+ and E- plants were estimated using the ‘specaccum’ function and the exact method. Beta-diversity indexes were estimated using the ‘betadiver’ function and the z index based on the Arrhenius species-area model (Koleff et al., 2003). Differences in beta diversity among groups were determined by Tukey multiple comparisons. A CCA was made because the gradient length of the detrended correspondence analysis (DCA) was greater than four, which indicated an unimodal response (Lepš and Šmilauer, 2003). Taxa appearing in less than three plants were omitted for this analysis; as a result, 61 taxa remained. A forward selection procedure (ordistep function) was used to determine the subset of explanatory variables (*Epichloë* incidence, population, *Epichloë*: population) explaining most variation in root mycobiome. The statistical power of the analysis was assessed by Monte Carlo permutation tests (*n* = 999).

### Salt Tolerance of Fungal Isolates

A set of 46 fungal strains belonging to 20 of the most abundant genera isolated from FRP roots plus nine *Epichloë festucae* strains were analyzed to determine their salt tolerance *in vitro*. For each fungal strain a 6 mm diameter mycelial disk was placed in the center of 9 cm Petri plates with PDA containing three different concentrations of sodium chloride: 600 mM (equivalent to sea water concentration), 300 mM, and a control without NaCl. For each fungal strain and salt treatment three replicate plates were prepared. All plates were incubated at room temperature in the dark. The colony diameter was measured at two perpendicular axes when colonies in the fastest growing medium reached a diameter of 4–6 cm. The effect of salinity treatments on the radial growth of fungal colonies was assessed by means of a one-way ANOVA, and statistical significance of differences among means using Tukey’s test (*p* < 0.05).

### Extracellular Enzyme Activity

*In vitro* cellulase and amylase activity was analyzed for 43 strains belonging to some of the most abundant taxa. The production of cellulase was assayed using the method described by Sunitha et al. (2013) adapted to PDA plates. For each fungal strain a 6 mm diameter mycelial disk was placed in the center of a 9 cm. Petri plate and incubated for 5 days at 25°± 1°C in the dark. After incubation the plates were flooded with 0.2% (w/v) aqueous Congo Red, and distained with 1 M NaCl for 15 min. The presence of a clear zone surrounding the colony indicated cellulase activity. Amylase activity was assessed on PDA containing 2% (w/v) soluble starch. After incubation the plates were flooded for 15 min with a solution of 1% (w/v) iodine in 2% (w/v) potassium iodide. A clear zone surrounding the colony indicated amylase activity (Hankin and Anagnostakis, 1975).

### Inoculation of *Lolium perenne* Plants With Root Endophytes From FRP

To test whether FRP endophytes affect the growth of the grass *Lolium perenne* under salinity, plants were inoculated with three fungal strains belonging to some of the core taxa from FRP roots. A greenhouse experiment was conducted with a completely randomized design with 14 plant replicates for each fungal strain (*Periconia* S6, *Penicillium* E7, and *Diaporthe* S69) and salinity treatment (0 and 200 mM NaCl). Seeds of *Lolium perenne* cv. Tivoli (DLF, Denmark) were sown in 200 mL plastic pots filled with a substrate composed of seven parts of peat and perlite (1:1) previously sterilized at 80°C for 24 h, mixed with one part (v:v) of fungal inoculum. The fungal inoculum was a 4 week old culture of each fungus grown in autoclaved sugar beet pulp. Several seeds were sown in each pot, and thinned to four seedlings after emergence. Three weeks after germination, plants were watered with 0 or 200 mM NaCl during 3 weeks. Plants subject to the salinity treatment were watered with 50 and 100 mM NaCl on the first and third day respectively to avoid salt shock, and the 200 mM concentration was applied from day 5 onward. After 3 weeks of salt treatment the plants were harvested.

Five replicates of each treatment (salt and fungal strain) were analyzed for K and Na concentration by inductively coupled plasma atomic emission spectroscopy (ICP-OES, Varian 720-ES). Previously, dried plant samples were calcined at 450°C for 8 h, and ashes dissolved in HCl:HNO_3_:H_2_O (1:1:8).

A two-way ANOVA was made to determine the effects of salt treatment and fungal strain on shoot biomass, K and Na concentrations, and differences between means were assessed using Tukey’s test (*p* < 0.05). The success of the inoculation was determined after harvest by the reisolation of the inoculated fungi from surface disinfected roots, using the method above explained.

## Results

### Endophyte Isolation

After plating 3150 root fragments on culture media, a total of 2324 fungal isolates were obtained, ranging from 355 to 578 among populations (Table 1). Most isolates emerged in the first 5 days after the placement of the roots on plates. Isolates were obtained from 73.8% of the root fragments plated. All sampled plants harbored fungi in their roots, and on average, 21 isolates were obtained from the roots of each plant.

**Table 1:** Incidence of *Epichloë* and fungal species richness in roots of *Festuca rubra* subsp. *pruinosa* at five populations from marine cliffs in Northern Spain.

Population	Number of plants analyzed	Incidence of *Epichloë festucae* (%)	Root mycobiota			
			Number of isolates obtained	Colonization^1^	Number of fungal species	Fungal species per plant
TDH	21	57.1	471	74.8	34	1.62
CED	19	68.4	355	62.3	46	2.42
EDB	22	77.3	473	71.7	47	2.47
CDP	20	20.0	447	74.5	59	2.57
SPR	23	100.0	578	83.8	46	2.19
Total/mean	105	65.7	2324	73.8	135	1.29

*^1^Percentage of root pieces from which fungi emerged into growth medium.*

*Epichloë festucae* was isolated from leaves of 65.7% of the plants. Its incidence among populations ranged from 20.0 to 100.0% (Table 1).

### Identification of Fungal Isolates and Taxonomic Structure

When the isolates of each population were grouped according to morphotypes, the TDH isolates were classified into 177 morphotypes, CED in 142, EDB in 125, SPR in 137, and those from CDP in 107.

**FIGURE 1:**
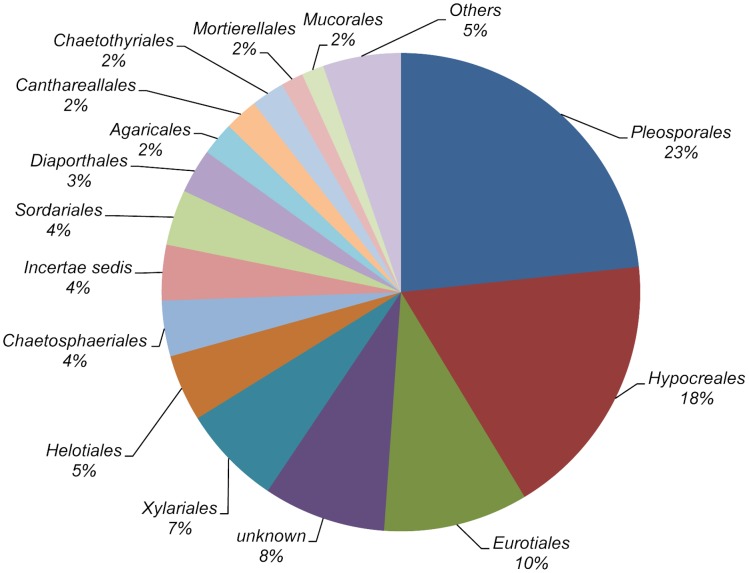
Distribution of fungal taxa from roots of *Festuca rubra* subsp. *pruinosa* plants from marine cliffs in northern Spain according to orders.

Nucleotide sequences were obtained from one or more isolates of each morphotype. As a result, 502 ITS1-5.8S-ITS2 nucleotide sequences were obtained, and those differing in homology by less than 3% were considered to belong to the same taxon. After this clustering process, 138 different sequences remained. These sequences were used to interrogate the UNITE sequence database, and as a result 135 fungal taxa were identified (Supplementary Table S1). Twenty-three taxa were identified to a species rank, 69 to genus rank and the remaining 43 were assigned to an order, class, family or division (Table 2). All the taxa could be assigned to 64 different fungal genera, 96% of them within the Ascomycota. Pleosporales, Hypocreales, and Eurotiales were the most representative orders, in terms of the number of taxa (23, 18, and 10%, respectively). The remaining orders were marginally represented (Figure 1). Among plant populations the number of fungal taxa ranged from 34 to 59 (Table 1).

**Table 2:** Core and abundant fungal species isolated from surface sterilized roots of *Festuca rubra* subsp. *pruinosa* at five populations from marine cliffs in northern Spain.

Strain	Taxon	Identity to closest match (%)	ITS sequence accession number	Order	Incidence in plants (%)	Number of populations
T150	*Fusarium oxysporum*	100	MH578626	Hypocreales	57.1	5
EB4	*Diaporthe* sp. A	100	MH578627	Diaporthales	54.3	5
C29	*Fusarium* sp. A	100	MH626490	Helotiales	41.0	4
S75	Helotiales sp. A	100	MH626491	Helotiales	37.1	5
T105	*Drechslera* sp.	100	MH626492	Pleosporales	27.6	4
S132	*Slopeiomyces cylindrosporus*	100	MH626493	Magnaporthales	27.6	3
T120	*Penicillium* sp. F	100	MH626494	Eurotiales	20.0	5
S7	*Darksidea* sp.	99	MH628220	Pleosporales	17.1	3
T131	*Periconia macrospinosa*	100	MH628221	Pleosporales	16.2	3
T122	*Penicillium* sp. A	100	MH628222	Eurotiales	14.3	4
T16	*Alternaria* sp. A	99	MH628223	Pleosporales	13.3	3
S38	*Fusarium* sp. B	99	MH628224	Hypocreales	13.3	4
C2	*Dactylonectria alcacerensis*	100	MH628225	Hypocreales	13.3	4
E79	Helotiales sp. B	100	MH628226	Helotiales	11.4	3
T140	*Alternaria* sp. B	100	MH628227	Pleosporales	10.5	4
E74	*Lachnum* sp. A	99	MH628228	Helotiales	10.5	3
CP17	*Trichoderma* sp. B	100	MH628229	Hypocreales	10.5	2

*Only the taxa with an incidence in plants greater than 20% are listed. Supplementary Table S1 contains the complete list of 135 taxa identified.*

**FIGURE 2:**
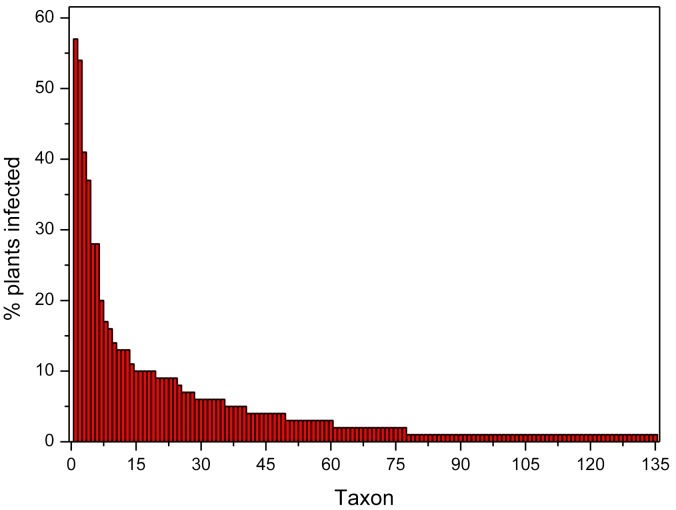
Rank-abundance plot showing the incidence in plants of each taxon identified in roots of *Festuca rubra* subsp. *pruinosa* plants from marine cliffs in northern Spain.

The distribution of the taxa according to their incidence can be visualized in the rank-abundance curve shown in Figure 2. Seven species occurred in more than 20% of the plants at three or more populations: *Fusarium oxysporum* (57.1%), *Diaporthe* sp. A (54.3%), *Fusarium* sp. A (40.9%), Helotiales sp. A (37.1%), *Slopeiomyces cylindrosporus* (27.6%), *Drechslera* sp. (27.6%), and *Penicillium* sp. F (20.0%) (Table 2). The identification of several *F. oxysporum* strains was confirmed by Martijn Rep and Maria Constantin (University of Amsterdam) by means of an analysis of their EF1α gene sequence. Because of their relatively high incidence within and among populations, these taxa could be considered as part of the core microbiome of FRP.

A second set of relatively abundant taxa were isolated from 10 to 20% of the plants, and at two or more populations (Table 2), these were *Darksidea* sp., *Periconia macrospinosa*, *Penicillium* sp. *A*, *Alternaria* sp. A, *Fusarium sp. B*, *Dactylonectria alcacerensis*, Helotiales sp. B, *Alternaria* sp. B, *Lachnum* sp. A and *Trichoderma* sp. B. The remaining 118 taxa were found in less than 10% of the plants and 58 of them were singletons, occurring in a single plant.

Some of most abundant taxa, like *Darksidea* sp.*, Periconia macrospinosa, Slopeiomyces cylindrosporus* and *Drechslera* sp., belong to the group of fungi known as dark septate endophytes (DSE). Fungi from the DSE group present some particular morphological characteristics, such as septated and melanized hyphae. These characteristics were observed in hyphae from two strains of Helotiales sp. A under the light microscope. Therefore, Helotiales sp. A also seems to belong to the DSE.

**FIGURE 3:**
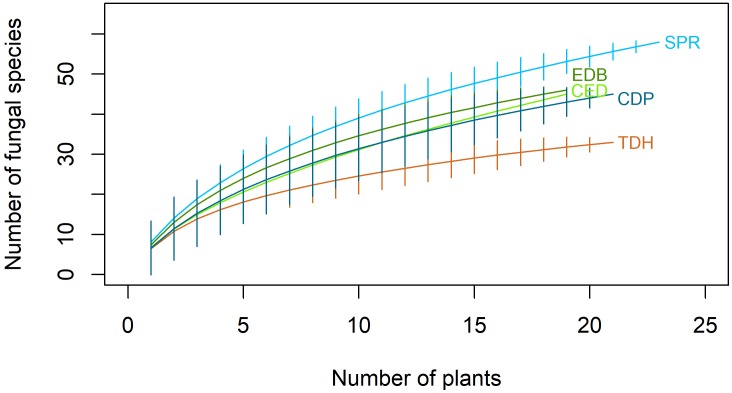
Species accumulation curves for fungal species isolated from roots of *Festuca rubra* subsp. *pruinosa* at five populations from marine cliffs in northern Spain. TDH, Torre de Hércules; CED, Cedeira; EDB, Estaca de Bares; SPR, San Pedro de la Rivera; CDP, Cabo de Peñas.

All populations produced non-asymptotic species accumulation curves, suggesting that increased sampling effort would reveal new fungal species (Figure 3). The Chao and Bootstrap estimators of the maximum number of species did not approach an horizontal asymptote, what made them unreliable estimators for this particular case.

### Effect of *Epichloë festucae* on Root Endophytic Fungal Communities

In the set of 105 plants analyzed, 69 were infected by *Epichloë festucae* (E+) and 36 were not (E-). Out of the 135 fungal species identified in all plants, 52 were exclusive of E+ plants, 29 of E- plants, and 54 occurred in both.

The ANOVA showed that neither the presence of *Epichloë* nor population had a significant effect on species richness (*F* = 1.999; *p* = 0.276 and *F* = 1.626; *p* = 0.174 respectively). The beta diversity index showed a similar trend, no significant differences were found between E+ and E- plants (*p* = 0.989) or among populations (*p* = 0.377 for all pairwise comparisons). The values of the Shannon diversity index (H’) were relatively high, but similar for E+ and E- plants (Table 3).

**Table 3:** Fungal species richness and diversity in roots of *Festuca rubra* subsp. *pruinosa* plants infected (E+) or not infected (E-) by *Epichloë festucae* at five populations in marine cliffs.

Factor		Number of plants analyzed	Species per plant	ß diversity (Kolleff)	H’ Shannon
*Epichloë*	E+	69	7.19 ± 2.63	0.59 ± 0.07	4.04
	E-	36	7.08 ± 3.11	0.59 ± 0.08	3.90
Population	TDH	21	6.52 ± 1.91	0.51 ± 0.10	3.13
	CED	19	6.84 ± 2.59	0.50 ± 0.10	3.32
	EDB	22	7.47 ± 2.55	0.56 ± 0.10	3.48
	CDP	20	6.67 ± 3.45	0.55 ± 0.10	3.59
	SPR	23	8.17 ± 3.04	0.55 ± 0.05	3.43

**FIGURE 4:**
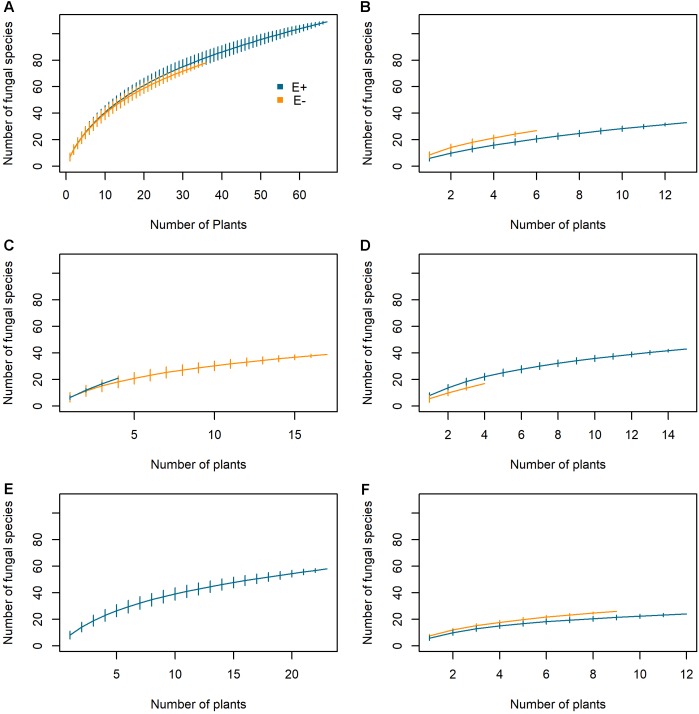
Species accumulation curves of root mycobiota in *Epichloë festucae* infected (E+) and non-infected (E–) plants of *Festuca rubra* subsp. *pruinosa* from five marine cliff populations in northern Spain. **(A)** Whole plant set; **(B)** Cedeira; **(C)** Cabo de Peñas; **(D)** Estaca de Bares; **(E)** San Pedro de la Rivera; **(F)** Torre de Hércules.

Both E*+* and E- plants displayed similar species accumulation curves when the data from all five populations were pooled (Figure 4A). The species richness accumulated at 36 plants was 80.93 ± 5.24 for E+ plants and 72.03 ± 1.04 for E- plants. Within each population, we found small differences (both positive and negative) between E+ and E- plants (Figures 4B–F).

**FIGURE 5:**
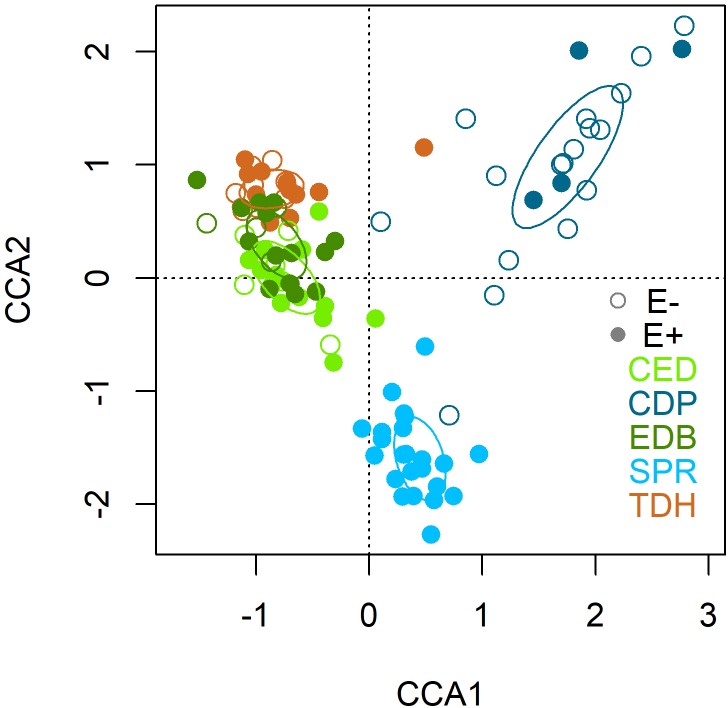
Canonical correspondence analysis (CCA) of the fungal endophyte community composition of roots of *Festuca rubra* subsp. *pruinosa* from marine cliffs according to the presence (E+) or absence (E–) of *Epichloë festucae*, and population (CED, Cedeira; CDP, Cabo de Peñas; EDB, Estaca de Bares; SPR, San Pedro de la Rivera; TDH, Torre de Hércules).

**FIGURE 6:**
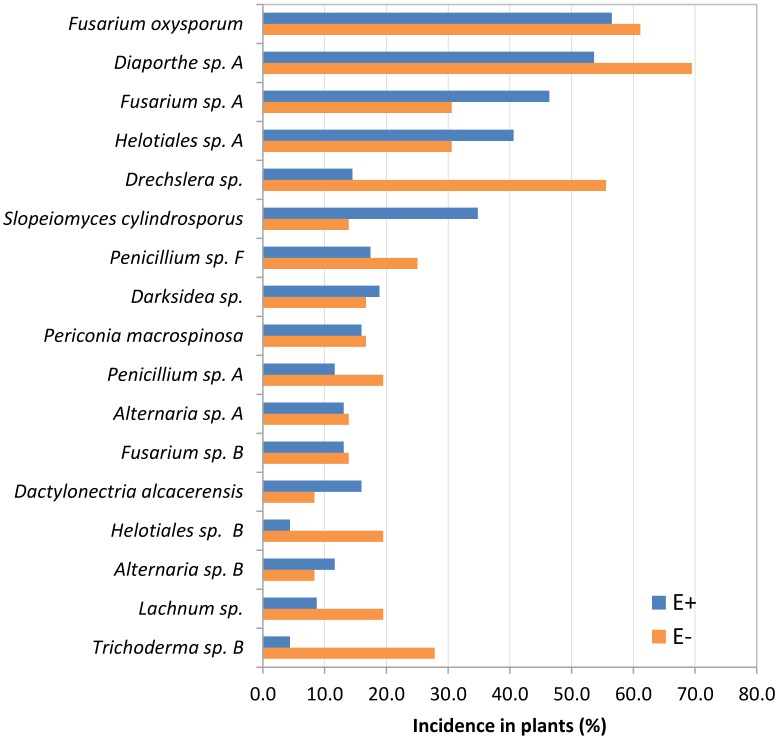
Incidence in plants of *Festuca rubra* subsp. *pruinosa* from marine cliffs infected (E+) and not infected (E–) by *Epichloë festucae* of root species that constitute the core and abundant classes of the culturable mycobiome.

The first two axes of the CCA were statistically significant (*p* = 0.001) and explained 35.18 and 29.36% of the variance. After the forward selection, only the variable population was finally included in the CCA and explained the 5.29% of the variation. The CCA biplot showed no clear separation between E+ and E- plants (Figure 5). However, there was a segregation among plant populations: the first axis clustered populations according to regions and separated the Asturian populations (CDP and SPR) from the Galician ones (TDH, CED and EDB); and the second axis segregated both Asturian populations, suggesting that the structure of the root mycobiota of these two populations differ between them and with respect to the Galician populations (Figure 5). All the core and the abundant taxa were present in both E+ and E- plants, although some species were more abundant in E+ (*Slopeiomyces cylindrosporus*) or in E- plants (*Drechslera* sp.) (Figure 6).

In terms of similarity of the fungal assemblages between pairs of populations, *J* values were higher between populations from the same region, 0.238 to 0.362 among Galician populations and 0.238 between Asturian populations, than between Galician and Asturian populations, which ranged from 0.095 to 0.193 (Table 4).

**Table 4:** Jaccard index of similarity (bold) and number of fungal species identified in roots of each pair of populations (italic) of *Festuca rubra* subsp. *pruinosa* plants from marine cliffs.

Population	TDH	CED	EDB	SPR	CDP
TDH	**1.000**	**0.238**	**0.362**	**0.095**	**0.147**
CED	*63*	**1.000**	**0.300**	**0.182**	**0.154**
EDB	*58*	*70*	**1.000**	**0.193**	**0.182**
SPR	*84*	*88*	*88*	**1.000**	**0.238**
CDP	*68*	*78*	*77*	*84*	**1.000**

### Salt Tolerance and Enzymatic Activity of Endophytic Fungi

The salt tolerance assay showed that fungal strains had three different types of response in terms of their radial growth. Most strains analyzed (71.8%) were halophilic, showing a statistically significant increase in radial growth in PDA plates containing NaCl respect to the control (Supplementary Table S2). The radial growth of 51.5% of these halophilic strains increased at both NaCl concentrations; that of 21.2% increased only in 600 mM NaCl, and that of 27.3% increased only in 300 mM NaCl. All nine *Fusarium* strains and four of the five *Diaporthe* sp. A strains tested were halophylic.

Some strains (6.5%) were halotolerant, not showing a significant difference in radial growth in 300 mM and 600 mM NaCl with respect to the control. Finally, 21.7% of the strains showed a radial growth decrease in culture media containing NaCl and were classified as halosensitive, 80.0% of these strains decreased only in 600mM NaCl, and the remaining 20.0% did it at both salt concentrations. Within taxa like *Diaporthe* sp. A, *Periconia macrospinosa* or *Penicillium* sp. F, some strains had different responses, i.e., *Diaporthe* strain S129 was halophilic and strain S69 halosensitive (Supplementary Table S2).

The nine *E. festucae* strains tested were halosensitive, all decreased in radial growth in the 600 mM medium (Table 5). Seven of them did not show a significant difference in radial growth with respect to the control at 300 mM NaCl.

**Table 5:** Radial growth of nine *Epichloë festucae strains* isolated from *Festuca rubra* subsp. *pruinosa* plants from marine cliffs in PDA plates with different NaCl concentrations.

Strain	Radial growth (cm)			Type of response	
	0 mM	300 mM	600 mM		
TDH1	2.12 ab	2.40 b	1.60 a	Halosensitive	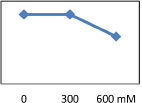
TDH11	1.97 ab	2.42 a	1.82 b	Halosensitive	
TDH3	2.67 a	2.60 a	1.62 b	Halosensitive	
CED6	2.67 a	2.17 ab	1.43 b	Halosensitive	
CED12	2.43 a	2.37 a	1.55 b	Halosensitive	
CED10	2.48 a	2.40 a	1.32 b	Halosensitive	
CED1	2.42 a	2.33 a	1.42 b	Halosensitive	
EDB9	2.37 a	1.25 b	0.67 c	Halosensitive	
EDB11	2.85 a	1.65 b	0.68 c	Halosensitive

*For each row different letters indicate significant differences at p < 0.05.*

Cellulase and amylase activities were assayed for 43 fungal strains (Table 6). Twenty three of these strains, including all tested strains of *Fusarium oxysporum, Penicillium* and Helotiales sp. A, showed cellulase activity *in vitro*. In contrast, none of the six *Diaporthe* sp. A strains tested was positive. Amylase activity was detected in only nine strains, including all four *Penicillium* strains tested.

**Table 6:** Cellulase and amylase activity in fungal strains isolated from roots *Festuca rubra* subsp. *pruinosa* plants from marine cliffs.

ID	Endophyte	Cellulase activity	Amylase activity
T16	*Alternaria* sp. *A*	-	++
C115	*Alternaria* sp. *B*	-	+
T90	*Codinaeopsis* sp.	++	-
C2	*Dactylonectria alcacerensis*	-	-
C1	*Darksidea* sp.	+	-
C7	*Darksidea* sp.	+	-
CP36	*Diaporthe* sp. A	-	-
EB4	*Diaporthe* sp. A	-	-
S129	*Diaporthe* sp. A	-	+
S32	*Diaporthe* sp. A	-	-
S69	*Diaporthe* sp. A	-	-
T18	*Diaporthe* sp. A	-	-
CP1	*Drechslera* sp.	-	-
E71	*Drechslera* sp.	-	-
T41	*Drechslera* sp.	-	-
T50	*Drechslera* sp.	-	-
CD8	*Epichloë festucae*	-	-
S13	*Fusarium* sp. A	+	-
T112	*Fusarium* sp. A	+	-
T6	*Fusarium* sp. A	+	-
C70	*Fusarium* sp. B	+	-
S38	*Fusarium* sp. B	+	+
CP3	*Fusarium oxysporum*	++	-
S10	*Fusarium oxysporum*	+	++
SP8	*Fusarium oxysporum*	+	-
T150	*Fusarium oxysporum*	+	-
E79	Helotiales sp. B	+++	-
S74	*Lachnum* sp.	-	-
C44	Helotiales sp. A	++	-
S75	Helotiales sp. A	++	-
T141	Helotiales sp. A	++	-
T29	Helotiales sp. A	+	-
T3	Helotiales sp. A	++	-
T114	*Penicillium* sp. F	++	+++
C13	*Penicillium* sp. A	+	+
E7	*Penicillium* sp. A	+++	+
T59	*Penicillium* sp. A	++	+
S6	*Periconia macrospinosa*	-	-
T131	*Periconia macrospinosa*	-	-
C43	*Slopeiomyces cylindrosporus*	-	-
S5	*Slopeiomyces cylindrosporus*	-	-
T70	*Slopeiomyces cylindrosporus*	-	-
CP17	*Trichoderma* sp. B	++	-

*(-) No enzymatic activity; (+) Slight activity; halo < 3 mm. (++) Moderate activity; halo < 5 mm. (+++) High activity; halo > 5 mm.*

### Effect of FRP Endophytes on Growth of *Lolium perenne*

**FIGURE 7:**
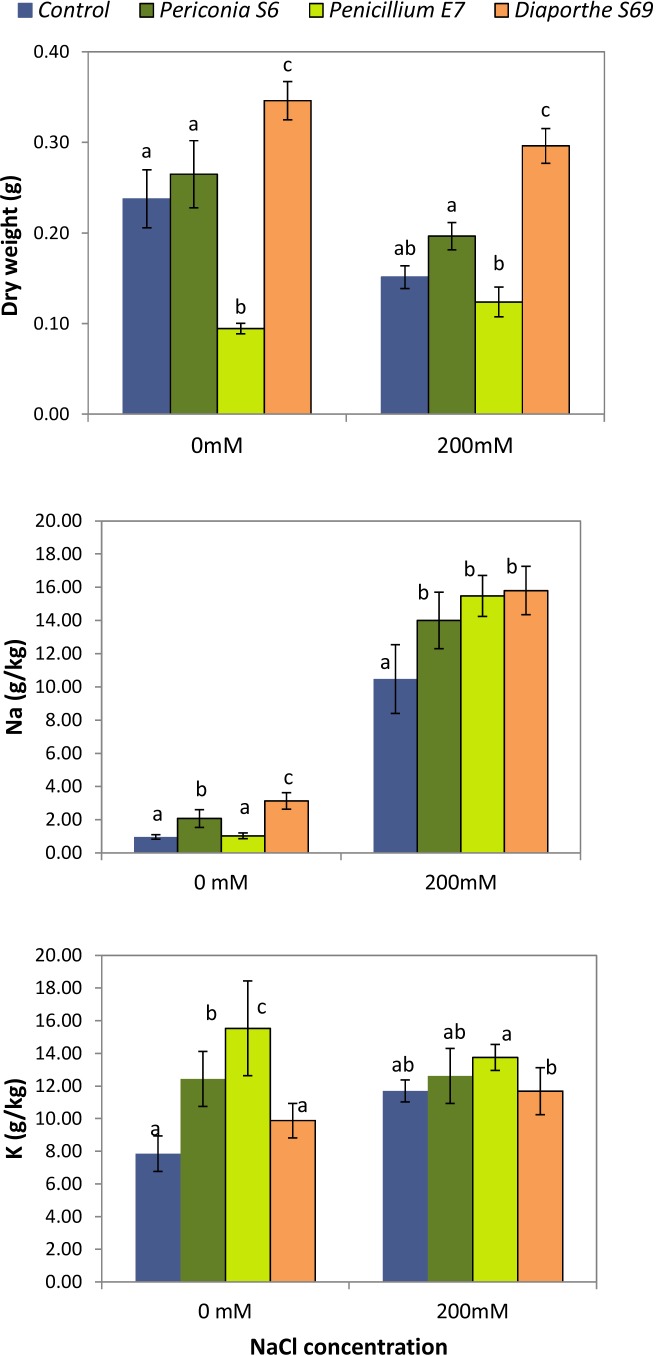
Effect of inoculation with strains *Periconia* S6, *Penicillium* E7 and *Diaporthe* S69, isolated from *Festuca rubra* subsp. *pruinosa*, on dry matter production, and Na and K content of *Lolium perenne* plants watered with 0 mM and 200 mM NaCl. For each NaCl concentration, different letters indicate significantly different means (*p* < 0.05).

A two-way ANOVA showed a significant effect of salinity (*p* = 0.004; $\bar{X}$_control_= 0.236 g, $\bar{X}$_NaCl_= 0.192 g), endophyte inoculated (*p* < 0.001; $\bar{X}$_control_= 0.194 g, $\bar{X}$*_Periconia_* = 0.231 g, $\bar{X}$*_Penicillium_* = 0.109_,_ $\bar{X}$*_Diaporthe_* = 0.321 g), and their interaction (*p* = 0.034; Figure 7) on dry matter production of *L. perenne*. Plants inoculated with *Diaporthe* S69, a *Diaporthe* sp. A strain, showed a significant increase in biomass production with respect to the uninoculated control plants in both watering treatments: 31.3% in tap water and 48.9% under saline irrigation (Figure 7). The plants inoculated with *Periconia* S6 had greater biomass in both watering treatments, but the difference respect to the controls was not significant. In contrast, plants inoculated with *Penicillium* E7 did not show visual symptoms of stress such as dry leaves, but showed a significant decrease in biomass production under the tap water treatment; in the salinity treatment the difference in biomass was not significant with respect to uninoculated plants. In addition, the biomass of plants inoculated with *Penicillium* E7 did not differ between tap water and salinity treatments.

Sodium was significantly affected by salt (*p* < 0.001), endophyte inoculated (*p* < 0.001) and their interaction (*p* = 0.002). Inoculated plants with *Periconia* S6 and *Diaporthe* S69 strains had greater Na than controls under tap water treatment (Figure 7). When plants were salt irrigated, the increase in Na was greater in plants inoculated with E7, S6 or S69 strains than in control plants. Potassium content was significantly affected by salt (*p* = 0.038), endophyte inoculated (*p* < 0.001) and their interaction (*p* = 0.003). Inoculated plants with E7, S6 or S69 strains had significantly greater K concentration than controls at water treatment (Figure 7). At salt treatment, plants inoculated with *Penicillium* E7 had the greatest K content.

After the harvest, root fragments of *Lolium perenne* were plated on culture media and the fungal isolates obtained were identified through morphological characteristics as the endophytes inoculated into the plants. The reisolation of these fungi indicated their compatibility with *L. perenne* and the success of plant inoculation.

## Discussion

### The Core Microbiome of *Festuca rubra* subsp. *pruinosa*

The roots of *Festuca rubra* subsp. *pruinosa* were found to be a niche containing numerous fungal species, an assemblage of 135 culturable species was identified. This magnitude is not unusual in surveys of the mycobiota of grasses (Sánchez Márquez et al., 2012), but the high incidence of seven species that were present in more than 20% of the plants, and in several populations is remarkable. These species were *Fusarium oxysporum*, *Diaporthe* sp. A, *Fusarium* sp. A, Helotiales sp. A, *Drechslera* sp., *Slopeiomyces cylindrosporus*, and *Penicillium* sp. F. In particular, *Fusarium oxysporum* and *Diaporthe* sp. A occurred in more than 50% of the plants, and at all five populations examined. Those seven fungal species seem to be components of the core microbiome of FRP, because they are shared by a significant number of plants, and occur at different populations (Shade and Handelsman, 2012). It is not common to find a group of fungal species with such high incidence within and among plant populations. Using similar methodology, as well as culture independent methods, no more than two or three species with an incidence greater than 20% were found in surveys of other grasses (Sánchez Márquez et al., 2008, 2010; Ofek-Lalzar et al., 2016; Zhong et al., 2018). In addition, dominant species reported in several taxa of inland grasses, such as *Cladosporium* or *Epicoccum*, were absent from FRP plants (Peláez et al., 1998; Sánchez Márquez et al., 2012; Ofek-Lalzar et al., 2016).

Two of the core taxa of FRP belonged to the genus *Fusarium*. Although this genus is best known due to important pathogens of numerous agricultural species, it is also one of the most commonly isolated genera of endophytes from grasses and other plants (Vázquez de Aldana et al., 2013; Martins et al., 2016; Lofgren et al., 2018). Research on endophytic *Fusarium* has shown that some strains can improve the salinity tolerance of their host plants (Rodriguez and Redman, 2008; Redman et al., 2011). Furthermore, *F. oxysporum* strains obtained from FRP plants in this study were found to protect tomato plants against a pathogenic strain of *F. oxysporum* f.sp. *lycopersici* (Constantin et al., 2017).

The genus *Diaporthe* contains numerous species that behave as endophytes or pathogens, and in some cases as both, depending on the host plant species (Gomes et al., 2013). *Diaporthe* sp. A is a main component of the core microbiome of FRP, and species of this genus have also been reported as dominant components of the microbiome of olive and other plants (Martins et al., 2016; Noriler et al., 2018). Regarding mutualism, *Diaporthe* strains originally isolated from wild plant species promoted the growth of rice and tritordeum (Yang et al., 2015; Zabalgogeazcoa et al., 2018).

Our work revealed that associations between DSE and FRP roots are common in sea cliffs. Some of the core and most abundant taxa, such as *Darksidea* sp.*, Periconia macrospinosa, Slopeiomyces cylindrosporus* and *Drechslera* sp., were previously reported as DSE in other grasses (Hornby et al., 1977; Knapp et al., 2012, 2015; Siless et al., 2018). In addition, Helotiales sp. A also seems to be a DSE because its hyphae had characteristics of this group, and other members of the Helotiales (i.e., *Phialocephala fortinii*) are recognized as DSE (Sieber and Grünig, 2013; Ridout et al., 2017). DSE colonize roots of plants communities in different habitats, and some authors hypothesized that these fungi can play an important role in plant adaptation to abiotic stress conditions, especially drought (Porras-Alfaro et al., 2008; Knapp et al., 2015). However, in spite of their abundance in nature, there is still uncertainty about the ecological significance of plant-DSE symbioses (Mandyam and Jumpponen, 2014).

Given the characteristics of the FRP habitat, strains from taxa belonging to the core microbiome of FRP are excellent candidates to test their possible role in host plant adaptation to salinity. Habitat-adapted symbiosis is a phenomenon which occurs when plants establish relationships with symbionts which enhance their adaptation to a particular stress factor present in their habitat (Rodriguez and Redman, 2008). Whether this occurs in the plant-endophyte systems here described would require inoculation of FRP seedlings and evaluation of plant performance parameters under salinity stress. The search for endophytes from the core microbiome of wild plants adapted to unhospitable habitats has produced interesting solutions for the improvement of stress tolerance on agronomic crops (Redman et al., 2011; Ali et al., 2018).

Because of our research interest in culturable fungi, and the isolation methods used, components of the plant microbiome such as bacteria or non-culturable fungi were not identified in this survey. Members of these groups could have an important role in the adaptation of FRP plants to marine cliffs. For instance, symbioses with arbuscular mycorrizal fungi (AMF) can contribute to plant growth and protection under environmental stress (Lenoir et al., 2016). Symbiotic associations with AMF have been reported for some *Festuca* species (i.e., Dalpé and Aiken, 1998; Santos et al., 2006), but their presence and effects in FRP were not studied, and deserve attention.

In this work, about 72% of the fungal strains from FRP roots were classified as halophilic, their radial growth *in vitro* increased in the presence of NaCl. This category included some species of the core microbiome of FRP, like *Diaporthe* sp. A, *Fusarium oxysporum*, *Fusarium* sp. A, and Helotiales sp. A. In contrast*, E. festucae* showed a halosensitive response. The life cycle of this fungus which colonizes the intercellular space of aerial tissues and is seed transmitted, can be completely endophytic. Thus, host plants protect the fungus from the harmful saline environment. However, other fungal species which spend a part of their life cycle outside of their plant hosts might benefit from being halotolerant.

Cellulase or amylase enzymatic activity *in vitro* was detected in some of the core taxa, such as *Fusarium oxysporum*, Helotiales sp. A and *Penicillium* sp. F. These enzymes degrade cellulose and starch to soluble sugars such as glucose, cellobiose, and other oligomers which can be readily absorbed by plant roots (Carroll et al., 1983). Considering that FRP plants grow in rock fissures where soil and nutrients are very scarce, fungi with these enzymatic activities could have a role recycling nutrients from dead roots. However, these two enzymatic activities were not detected in cultures of *Slopeiomyces cylindrosporus*, a fungus with saprobic capability (Hornby et al., 1977), and cellulase activity was absent form *Diaporthe* sp. A strains. This result could be due to non-induction of these enzymes in the culture medium used, because both fungal strains grew well as saprobes in a beet pulp medium, rich in carbohydrate and protein, which was used to prepare inoculum for plant inoculations.

### Potential of FRP Endophytes for Plant Improvement

Knowledge about the role of endophytic fungi on plant adaptation to salinity stress is important because the world surface of saline soils is increasing, producing economic losses in crops (Munns and Gilliham, 2015). *Diaporthe* sp. A strain S69 improved the growth of plants of *Lolium perenne*, an important forage grass, in the presence and absence of salinity stress. On average, plants inoculated with *Diaporthe* S69 produced 31% more aerial biomass than the uninoculated controls under normal conditions, and 49% more under salinity stress. Similarly, fungal endophytes such as *Piriformospora indica*, *Fusarium culmorum*, or *Penicillium minioluteum* can alter physiological processes and improve tolerance to salt stress in agricultural crop species (Baltruschat et al., 2008; Khan et al., 2011; Redman et al., 2011).

One of the indirect consequences of salinity is an enrichment of Na and deficiency of K in plant cells, caused by the competition between Na and K, that have similar ionic radii and ion hydration energies (Munns and Tester, 2008). We found that *L. perenne* plants inoculated with *Periconia* S6, *Penicillium* E7 and *Diaporthe* S69 strains accumulated significantly more K in aboveground tissues under the tap water treatment than uninoculated plants; this suggests that an enrichment of Na due to salinity might have been prevented by the increased K content present before the stress. Similar results were observed in grasses inoculated with *Aspergillus aculeatus* (Xie et al., 2017) suggesting that the maintenance of a high level of K may contribute the alleviation of the negative effect of sodium. A beneficial effect of K accumulation in plants has also been reported for associations with arbuscular mycorrhiza (Langenfeld-Heyser et al., 2007) and *Epichloë* spp. (Chen et al., 2018). It is important to point out that the increase in biomass of *L. perenne* plants inoculated with *Diaporthe* strain S69 occurred not only during salt treatment but also in the tap water treatment. This implies that the fungal effect improving plant growth was not a specific process induced by salinity. To study the effect of fungal strains on plant parameters which can be altered by endophytes to improve plant performance, such as phytohormones, photosynthetic capacity, nutrient absorption or antioxidant capability (Baltruschat et al., 2008; Redman et al., 2011; Leitão and Enguita, 2016) is a future objective of our research.

### Effect of *Epichloë festucae*, an Aboveground Tissue Endophyte, on Root Mycobiota

The incidence of *Epichlöe festucae* in FRP populations was 65.7%, a value very similar to that of 69% observed in a previous survey that included the same populations from Galicia (Zabalgogeazcoa et al., 2006). The relatively high incidence of *E. festucae* suggests that in an unhospitable habitat like sea cliffs, the costs of harboring a systemic symbiont could be compensated by mutualism. However, endophyte incidences closer to 100% could be expected under such circumstances. Whether natural selection favoring E+ plants, the efficiency of seed transmission, or a combination of both processes are involved in the prevalence rates of *Epichloë* observed in FRP populations is unknown, and deserves further study. Imperfect seed transmission (<100%) has been reported in other grass – *Epichloë* systems (Gundel et al., 2009). High incidence of *Epichloë festucae* in *Festuca rubra* populations has been reported in semiarid grasslands (70%) (Zabalgogeazcoa et al., 1999), or in the Scottish islands of St. Kilda (80%) (Bazely et al., 1997). In contrast, in Finland only 9 of 49 infected *F. rubra* populations had frequencies greater than 50% (Wali et al., 2007), and no plants harboring *Epichloë* were found in populations from subarctic regions of Canada (Santangelo and Kotanen, 2016).

In some grass-endophyte associations *Epichloë* species could play a key role in salt tolerance. In pot experiments *Epichloë coenophiala* increased the root biomass of tall fescue (*Schedonorus arundinaceous*) (Sabzalian and Mirlohi, 2010), and another *Epichloë* species increased the shoot and root biomass of wild barley (*Hordeum brevisubulatum*) under salinity stress (Song et al., 2015; Chen et al., 2018). In contrast, in FRP plants no significant effect of *Epichloë* on shoot dry weight was detected under salt treatment, although root growth or other parameters that could be affected by the presence of *E. festucae* under salinity were not analyzed (Zabalgogeazcoa et al., 2006). Nevertheless, in a stressful habitat like sea cliffs, environmental pressure on a holobiont might not necessarily affect an individual endophyte, but an assemblage where interactions among the plant host and the eukaryotic and prokaryotic microbiome components might be complex.

The presence of *Epichloë* in aboveground tissues of the host plant can affect underground processes by altering rhizospheric conditions that affect the density and activity of soil microorganisms (Omacini et al., 2012). This may result from endophyte effects on root exudates that can act as chemical attractants or repellents in the rhizosphere (Malinowski et al., 1998). For instance, phenolic compounds are microbial inhibitors, and they increase in roots due to the presence of *Epichloë* (Ponce et al., 2009; Vázquez de Aldana et al., 2011). The effect of *Epichloë* on arbuscular mycorrhizal fungi has been extensively studied, and reduction, promotion, and null effects have been reported (Omacini et al., 2006; Novas et al., 2012; Rojas et al., 2016). Our results indicate that *E. festucae* did not have a clear and significant effect on the composition of the core microbiome or other mycobiota from FRP roots, although changes in the abundance of some species were found. These results are in agreement with other studies where the presence of *Epichloë* did not alter fungal colonization in roots (Vandegrift et al., 2015; Slaughter and McCulley, 2016) or shoots (Zabalgogeazcoa et al., 2013). Nevertheless, Zhong et al. (2018) reported that the presence of *Epichloë* decreased the diversity of root-associated fungi in *Achnatherum inebrians* and changed the community composition. However, such changes were in fungal orders with an abundance lower than 10%, where the number of isolates of these taxa can be low.

## Conclusion

In conclusion, this study shows that numerous species of culturable fungi are associated to the roots of *Festuca rubra* subsp. *pruinosa* in its sea cliff habitat. Within this fungal assemblage of 135 species, a set of seven species occurred in a relatively high number of plants and locations, and those seem to be components of the core mycobiome of FRP: *Fusarium oxysporum*, *Diaporthe* sp. A, *Fusarium* sp. A, Helotiales sp. A, *Drechslera* sp., *Slopeiomyces cylindrosporus*, and *Penicillium* sp. F. Strains of these species are very promising candidates to study their role in the adaptation of FRP plants to salinity, a characteristic stress factor of their habitat. Furthermore, a *Diaporthe* strain belonging to the core taxa significantly improved the growth of *Lolium perenne* plants under normal and salinity stress conditions, showing the potential of the FRP core microbiome for the improvement of agricultural crops.

## Author Contributions

EP collected the plants, isolated and identified fungi, made the experiments, and analyzed the data. BA designed the experiments, participated in plant collection, and analyzed the data. LS made the statistical analyses. IZ supervised the research, helped to sample plants, designed the experiments and analyzed the data. EP, BA, and IZ wrote the article.

## Conflict of Interest Statement

The authors declare that the research was conducted in the absence of any commercial or financial relationships that could be construed as a potential conflict of interest.
