# Supplementary material for: A Survey of Culturable Fungal Endophytes From Festuca rubra subsp. pruinosa, a Grass From Marine Cliffs, Reveals a Core Microbiome
Source: Front Microbiol. 2019 Jan 16;9:3321. doi: 10.3389/fmicb.2018.03321 (PMC6343541; doi:10.3389/fmicb.2018.03321)

Supplementary Material

A survey of culturable fungal endophytes from *Festuca rubra* subsp. *pruinosa*, a grass from marine cliffs, reveals a core microbiome

Eric Pereira[1], Beatriz Vázquez de Aldana[1] Leticia San Emeterio[2], and Iñigo Zabalgogeazcoa[1]

[1]Institute of Natural Resources and Agrobiology of Salamanca (IRNASA-CSIC), Salamanca, Spain

[2] Research Institute on Innovation & Sustainable Development in Food Chain (ISFood), Universidad Pública de Navarra, Pamplona, Spain

*** Correspondence:** i.zabalgo@irnasa.csic.es

Supplementary Table 1. Taxonomic identification of fungi isolated from surface sterilized roots of *Festuca rubra* subsp. *pruinosa* at five populations from marine cliffs in northern Spain.

Strain	Taxon	Identity to closest match (%)	ITS sequence accession number	Order	Incidence in plants (%)	Number of Populations
T150	*Fusarium oxysporum*	100	MH578626	Hypocreales	57.14	5
EB4	*Diaporthe* sp. A	100	MH578627	Diaporthales	54.29	5
C29	*Fusarium* sp. A	100	MH626490	Hypocreales	40.95	4
S75	*Helotiales* sp. A	100	MH626491	Helotiales	37.14	5
T105	*Drechslera* sp.	100	MH626492	Pleosporales	27.62	4
S132	*Slopeiomyces cylindrosporus*	100	MH626493	Magnaporthales	27.62	3
T120	*Penicillium* sp. F	100	MH626494	Eurotiales	20.00	5
S7	*Darksidea* sp.	99	MH628220	Pleosporales	17.14	3
T131	*Periconia macrospinosa*	100	MH628221	Pleosporales	16.19	3
T122	*Penicillium* sp. A	100	MH628222	Eurotiales	14.29	4
T16	*Alternaria* sp. A	99	MH628223	Pleosporales	13.33	3
S38	*Fusarium* sp. B	99	MH628224	Hypocreales	13.33	4
C2	*Dactylonectria alcacerensis*	100	MH628225	Hypocreales	13.33	4
E79	*Helotiales* sp. B	100	MH628226	Helotiales	11.43	3
T140	*Alternaria* sp. B	100	MH628227	Pleosporales	10.48	4
E74	*Lachnum* sp. A	99	MH628228	Helotiales	10.48	3

Strain	Taxon	Identity to closest match (%)	ITS sequence accession number	Order	Incidence in plants (%)	Number of Populations
CP17	*Trichoderma* sp. B	100	MH628229	Hypocreales	10.48	2
S115	*Alternaria* sp. C	98	MH633916	Pleosporales	9.52	4
T90	*Chaetosphaeriaceae* sp. A	99	MH633917	Chaetosphaeriales	9.52	2
T24	*Agaricales* sp.	100	MH633918	Agaricales	8.57	4
S4	*Plectosphaerella cucumerina*	100	MH633919	Incertae sedis	8.57	3
E18	*Pleosporales* sp. C	100	MH633920	Pleosporales	8.57	3
S8	*Pleosporales* sp. F	100	MH633921	Pleosporales	8.57	2
T107	*Sarocladium strictum*	100	MH633922	Incertae sedis	8.57	3
T174	*Helotiales* sp. C	100	MH633923	Helotiales	7.62	3
S113	*Ceratobasidium* sp. A	100	MH633924	Canthareallales	6.67	2
CP15	*Helotiales* sp. D	99	MH633925	Helotiales	6.67	1
S59	*Pleosporales* sp. B	100	MH633926	Pleosporales	6.67	3
S50	*Alternaria* sp. D	100	MH633927	Pleosporales	5.71	2
E35	*Chaetomium novozelandicum*	99	MH633928	Sordariales	5.71	1
C89	*Fusarium* sp. D	100	MH633929	Hypocreales	5.71	2
E58	*Lachnum* sp. B	99	MH633930	Helotiales	5.71	3
T176	*Marasmius* sp. A	99	MH633931	Agaricales	5.71	3
S100	*Nemania diffusa*	100	MH633932	Xylariales	5.71	2
E20	*Pyrenochaetopsis* sp.	99	MH633933	Pleosporales	5.71	2
C22	*Dichotomopilus* sp. A	99	MH633934	Sordariales	4.76	3
CP69	*Ilyonectria* sp. B	100	MH633935	Hypocreales	4.76	2
S94	*Mortierella* sp. A	100	MH633936	Mortierellales	4.76	2
S33	*Diaporthe* sp. B	100	MH633937	Diaporthales	4.76	1
CP74	*Dichotomopilus* sp. B	99	MH633938	Melanosporales	4.76	1
S104	*Chaetosphaeriales* sp. A	100	MH633939	Chaetosphaeriales	3.81	2
T165	*Clohesyomyces* sp.	99	MH633940	Pleosporales	3.81	1
E17	*Clonostachys* sp.	100	MH633941	Hypocreales	3.81	2
CP23	*Ascomycota* sp. B	93	MH633942	Hypocreales	3.81	1
E14	*Ilyonectria* sp. A	99	MH644805	Hypocreales	3.81	1
T25	*Microdochium* sp. A	99	MH633943	Xylariales	3.81	2
CP56	*Pleosporales* sp. I	92	MH633944	Pleosporales	3.81	1
CP88	*Sordariomycetes* sp.	99	MH633945	unknown	3.81	2
T96	*Trichoderma* sp. A	99	MH633946	Hypocreales	3.81	3
T74	*Absidia* sp.	99	MH633947	Mucolares	2.86	2

Strain	Taxon	Identity to closest match (%)	ITS sequence accession number	Order	Incidence in plants (%)	Number of Populations
S68	*Exophiala pisciphila*	100	MH633948	Chaetothyriales	2.86	3
S78	*Fusarium* sp. G	99	MH633949	Hypocreales	2.86	1
S19	*Fusarium* sp. H	100	MH633950	Hypocreales	2.86	1
CP59	*Marasmius tricolor*	100	MH633951	Agaricales	2.86	1
E2	*Penicillium* sp. E	99	MH633952	Eurotiales	2.86	1
S124	*Penicillium* sp. J	100	MH633953	Eurotiales	2.86	1
S107	*Periconia* sp.	100	MH633954	Pleosporales	2.86	1
S133	*Preussia* sp. B	97	MH633955	Pleosporales	2.86	1
T13	*Thozetella* sp.	99	MH633956	Chaetosphaeriales	2.86	2
T138	*Umbelopsis* sp.	99	MH633863	Mucolares	2.86	2
S87	*Acrocalymna* sp.	100	MH633864	Pleosporales	1.90	2
T39	*Ilyonectria robusta*	100	MH633865	Hypocreales	1.90	1
T143	*Ophiosphaerella* sp. A	99	MH633866	Pleosporales	1.90	2
CP14	*Paraconiothyrium* sp.	100	MH633867	Pleosporales	1.90	1
T111	*Paraphaeosphaeria* sp. A	99	MH633868	Pleosporales	1.90	1
S76	*Paraphaeosphaeria* sp. C	100	MH633869	Pleosporales	1.90	1
E45	*Penicillium* sp. G	98	MH633870	Eurotiales	1.90	1
S116	*Penicillium* sp. I	99	MH633871	Eurotiales	1.90	1
CP81	*Penicillium* sp. K	99	MH633872	Eurotiales	1.90	1
S129	*Diaporthe* sp. C	100	MH633873	Diaporthales	1.90	1
C66	*Diaporthe* sp. D	99	MH633874	Diaporthales	1.90	2
T153	*Pleosporales* sp. A	99	MH633875	Pleosporales	1.90	1
E61	*Clavicipitaceae* sp. A	100	MH633876	Hypocreales	1.90	1
S51	*Ceratobasidium* sp. B	99	MH633877	Canthareallales	1.90	1
CP51	*Talaromyces wortmannii*	100	MH633878	Eurotiales	1.90	1
T129	*Talaromyces* sp. A	100	MH633879	unknown	1.90	1
S102	*Ascomycota* sp. E	100	MH633880	unknown	1.90	1
S46	*Acrostalagmus luteoalbus*	100	MH633881	Hypocreales	0.95	1
C42	*Arthrinium* sp.	100	MH633882	Xylariales	0.95	1
S89	*Aspergillus nomius*	100	MH633883	Eurotiales	0.95	1
CP34	*Aureobasidium pullulans*	100	MH633884	Dothideales	0.95	1
CP11	*Bartalinia* sp.	99	MH633885	Amphisphaeriales	0.95	1
E48	*Boeremia exigua*	99	MH633886	Pleosporales	0.95	1
S80	*Capnodium* sp.	99	MH633887	Capnodiales	0.95	1

Strain	Taxon	Identity to closest match (%)	ITS sequence accession number	Order	Incidence in plants (%)	Number of Populations
C48	*Ceratobasidiaceae* sp. A	99	MH633888	Canthareallales	0.95	1
S72	*Dichotomopilus funicola*	100	MH633889	Sordariales	0.95	1
C33	*Chaetomium* sp. C	99	MH633890	Sordariales	0.95	1
C52	*Chaetosphaeriales* sp. B	95	MH633891	Chaetosphaeriales	0.95	1
S125	*Tolypocladium* sp.	99	MH633892	Hypocreales	0.95	1
T87	*Cryptosporiopsis* sp.	99	MH633893	Helotiales	0.95	1
E49	*Hypocreales* sp.	99	MH633894	Hypocreales	0.95	1
C9	*Cylindrocarpon* sp.	99	MH633895	Hypocreales	0.95	1
S63	*Exophiala* sp.	100	MH633896	Chaetothyriales	0.95	1
T155	*Fusarium* sp. F	100	MH633897	Hypocreales	0.95	1
CP19	*Fusarium* sp. I	99	MH633898	Hypocreales	0.95	1
S127	*Gliomastix murorum*	99	MH633899	Hypocreales	0.95	1
C95	*Xylariales* sp. A	100	MH633900	Xylariales	0.95	1
E38	*Microdochium* sp. C	97	MH644806	Xylariales	0.95	1
C90	*Microdochium* sp. D	99	MH633901	Xylariales	0.95	1
S135	*Mortierella* sp. B	100	MH633902	Mortierellales	0.95	1
T166	*Nemania serpens*	99	MH633903	Xylariales	0.95	1
C54	*Neopestalopsis clavispora*	100	MH633958	Xylariales	0.95	1
S28	*Ophiosphaerella* sp. B	100	MH633959	Pleosporales	0.95	1
CP58	*Paraphaeosphaeria* sp. B	100	MH633960	Pleosporales	0.95	1
S84	*Penicillium* sp. H	100	MH633961	Eurotiales	0.95	1
C16	*Pleosporales* sp. D	99	MH633962	Pleosporales	0.95	1
C5	*Pleosporales* sp. E	97	MH633963	Pleosporales	0.95	1
C104	*Pleosporales* sp. G	98	MH633964	Pleosporales	0.95	1
C63	*Pleosporales* sp. H	99	MH633965	Pleosporales	0.95	1
E70	*Pleosporales* sp. J	99	MH633966	Pleosporales	0.95	1
C8	*Sporormiella australis*	99	MH633967	Pleosporales	0.95	1
S25	*Preussia* sp. A	97	MH633968	Pleosporales	0.95	1
C32	*Acrocalymma vagum*	99	MH633969	Incertae sedis	0.95	1
S110	*Scytalidium* sp.	99	MH633970	Helotiales	0.95	1
S111	*Serendipita vermifera*	98	MH633971	Sebacinales	0.95	1
C56	*Sordaria* sp.	100	MH633972	Sordariales	0.95	1
CP95	*Sordariomycetes* sp. B	100	MH633973	unknown	0.95	1
E6	*Stemphylium vesicarium*	99	MH633974	Pleosporales	0.95	1

Strain	Taxon	Identity to closest match (%)	ITS sequence accession number	Order	Incidence in plants (%)	Number of Populations
CP86	*Talaromyces* sp. B	99	MH633975	Eurotiales	0.95	1
C49	*Trichoderma rossicum*	99	MH633976	Hypocreales	0.95	1
S93	*Trichoderma spirale*	99	MH633977	Hypocreales	0.95	1
T139	*Ascomycota* sp. A	97	MH633978	unknown	0.95	1
C28	*Ascomycota* sp. C	98	MH644807	unknown	0.95	1
S71	*Ascomycota* sp. D	99	MH633979	unknown	0.95	1
S105	*Ascomycota* sp. F	100	MH633980	unknown	0.95	1
E26	*Ascomycota* sp. G	99	MH633981	unknown	0.95	1
C27	*Ascomycota* sp. H	99	MH633982	unknown	0.95	1
E63	*Metarhizium* sp.	100	MH633983	unknown	0.95	1
CP66	*Clavicipitaceae* sp. B	98	MH633984	unknown	0.95	1
C67	*Ascomycota* sp. I	98	MH633985	unknown	0.95	1
S88	*Mortierella* sp. C	100	MH633986	unknown	0.95	1
E78	*Sordariomycetes* sp. C	99	MH633987	Glomerellales	0.95	1
CP96	*Xylaria* sp.	100	MH633988	Xylariales	0.95	1
CP45	*Xylariaceae* sp.	100	MH633989	Xylariales	0.95	1
E60	*Chaetosphaeriaceae* sp. B	99	MH633990	Chaetosphaeriales	0.95	1

Supplementary Table 2. Radial growth endophytic strains from *Festuca rubra* subsp. *pruinosa roots* grown in PDA plates with different NaCl concentrations. Different letters within each row indicate significant differences at *p*<0.05.

Strain	Taxon	Radial growth (cm) 0 mM	300 mM	600 mM	Type of response
E5	*Alternaria sp. A*	4.52 a	5.82 b	5.62 b	Halophilic
S41	*Alternaria sp. B*	3.83 a	5.58 b	5.05 c	Halophilic
S32	*Diaporthe sp.A*	5.92 a	7.50 b	6.77 c	Halophilic
CP36	*Diaporthe sp.A*	1.83 a	4.98 b	5.43 c	Halophilic
S129	*Diaporthe sp.C*	4.65 a	6.60 b	6.20 b	Halophilic
T95	*Fusarium sp. A*	4.52 a	6.42 b	5.63 c	Halophilic
S13	*Fusarium sp. A*	2.23 a	5.50 b	4.03 c	Halophilic
T112	*Fusarium sp. A*	3.20 a	7.43 b	6.17 c	Halophilic
C70	*Fusarium sp. B*	2.23 a	6.83 b	4.08 c	Halophilic
S38	*Fusarium sp. B*	4.90 a	6.87 b	6.57 b	Halophilic
CP8	*Fusarium oxysporum*	5.53 a	7.12 b	7.25 b	Halophilic
S10	*Fusarium oxysporum*	5.40 a	7.17 b	6.65 c	Halophilic
T150	*Fusarium oxysporum*	5.43 a	7.50 b	7.30 b	Halophilic
C44	*Helotiales sp. A*	4.98 a	6.10 b	6.30 b	Halophilic
E79	*Helotiales sp.B*	4.68 a	7.92 b	7.47 c	Halophilic
T59	*Penicillium sp. A*	3.67 a	7.02 b	6.93 b	Halophilic
CP17	*Trichoderma sp. B*	5.70 a	7.33 b	6.58 c	Halophilic

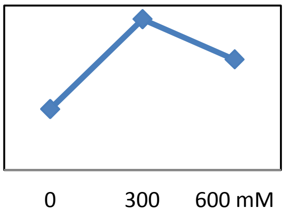

Strain	Taxon	0 mM	300 mM	600 mM	Type of response
S7	*Darksidea sp.*	2.18 a	2.25 a	3.02 b	Halophilic
T50	*Drechslera sp.*	6.67 a	6.55 a	7.30 b	Halophilic
T6	*Fusarium sp. A*	3.82 a	4.07 a	6.57 b	Halophilic
T114	*Penicillium sp. F*	5.60 a	5.73 a	7.32 b	Halophilic

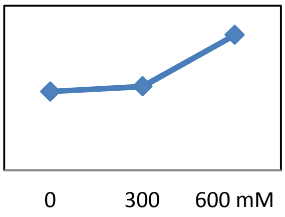

Strain	Taxon	0 mM	300 mM	600 mM	Type of response
EB4	*Diaporthe sp.A*	3.03 a	2.47 b	6.43 c	Halophilic
T18	*Diaporthe sp.A*	4.45 a	3.50 b	5.78 c	Halophilic
E7	*Penicillium sp. A*	6.65 a	6.07 b	7.20 c	Halophilic

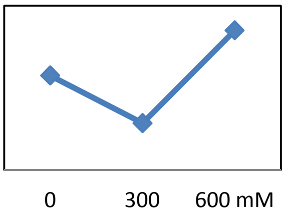

Strain	Taxon	Radial growth (cm)			Type of response
		0 mM	300 mM	600 mM	
S115	*Alternaria sp. B*	5.20 a	5.80 b	5.52 ab	Halophilic
T140	*Alternaria sp. C*	5.30 a	6.10 b	5.35 a	Halophilic
C66	*Diaporthe sp.A*	4.87 a	5.88 b	4.68 a	Halophilic
C13	*Penicillium sp. A*	4.47 a	4.72 b	4.38 a	Halophilic
T49	*Periconia macrospinosa*	4.92 a	5.97 b	5.00 a	Halophilic

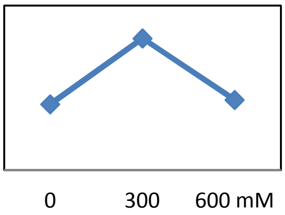

T16	*Alternaria sp. A*	4.07 a	5.13 b	3.02 c	Halophilic
C2	*Dactylonectria alcacerensis*	6.35 a	7.55 b	5.28 c	Halophilic
T141	*Helotiales sp. A*	5.83 a	6.65 b	5.07 c	Halophilic
E71	*Penicillium sp. A*	6.43 a	7.05 b	5.72 c	Halophilic

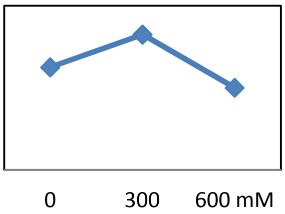

C1	*Darksidea sp.*	1.33 a	1.83 a	1.57 a	Halotolerant
E74	*Lachnum sp.*	1.65 a	1.58 a	1.42 a	Halotolerant
T128	*Helotiales sp. A*	6.35 a	6.13 a	5.80 a	Halotolerant

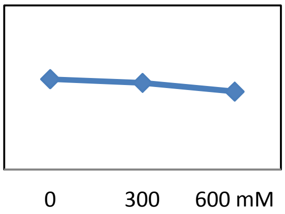

S69	*Diaporthe sp.A*	5.12 ab	6.01 a	4.45 b	Halosensitive
T90	*Codinaeopsis sp.*	5.03 a	5.03 a	3.43 b	Halosensitive
S75	*Helotiales sp.*A	6.23 a	6.08 a	4.82 b	Halosensitive
E54	*Penicillium sp. F*	5.48 a	5.71 a	5.03 b	Halosensitive
T131	*Periconia macroespinosa*	6.77 a	7.25 a	6.00 b	Halosensitive
S96	*Trichoderma sp. B*	6.15 a	6.23 a	4.66 b	Halosensitive
S5	*Slopeiomyces cylindrosporus*	7.50 a	7.25 a	1.18 b	Halosensitive
T70	*Slopeiomyces cylindrosporus*	3.62 a	3.78 a	2.42 b	Halosensitive

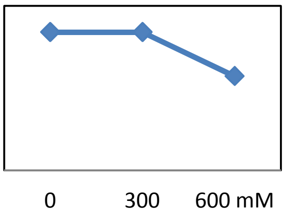

T41	*Drechslera sp.*	6.17 a	5.87 b	5.17 b	Halosensitive
C43	*Slopeiomyces cylindrosporus*	4.18 a	3.12 b	2.68 c	Halosensitive

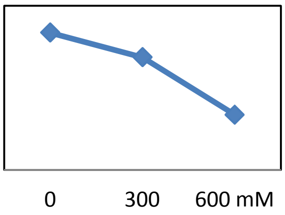

Supplement: Supplementary file 1 [file Data_Sheet_1.PDF]
